# Motion Estimation and Hand Gesture Recognition-Based Human–UAV Interaction Approach in Real Time

**DOI:** 10.3390/s22072513

**Published:** 2022-03-25

**Authors:** Minjeong Yoo, Yuseung Na, Hamin Song, Gamin Kim, Junseong Yun, Sangho Kim, Changjoo Moon, Kichun Jo

**Affiliations:** Department of Smart Vehicle Engineering, Konkuk University, 120, Neungdong-ro, Gwangjin-gu, Seoul 05029, Korea; dbalswjd57@konkuk.ac.kr (M.Y.); nysguy7@konkuk.ac.kr (Y.N.); shm5069@konkuk.ac.kr (H.S.); yondoo20@konkuk.ac.kr (G.K.); springboat@konkuk.ac.kr (J.Y.); kimsh85@konkuk.ac.kr (S.K.); cjmoon@konkuk.ac.kr (C.M.)

**Keywords:** human–UAV interaction, hybrid-based hand gesture recognition, hand-gesture-based recognition, IMU-based motion capture system, deep learning

## Abstract

As an alternative to traditional remote controller, research on vision-based hand gesture recognition is being actively conducted in the field of interaction between human and unmanned aerial vehicle (UAV). However, vision-based gesture system has a challenging problem in recognizing the motion of dynamic gesture because it is difficult to estimate the pose of multi-dimensional hand gestures in 2D images. This leads to complex algorithms, including tracking in addition to detection, to recognize dynamic gestures, but they are not suitable for human–UAV interaction (HUI) systems that require safe design with high real-time performance. Therefore, in this paper, we propose a hybrid hand gesture system that combines an inertial measurement unit (IMU)-based motion capture system and a vision-based gesture system to increase real-time performance. First, IMU-based commands and vision-based commands are divided according to whether drone operation commands are continuously input. Second, IMU-based control commands are intuitively mapped to allow the UAV to move in the same direction by utilizing estimated orientation sensed by a thumb-mounted micro-IMU, and vision-based control commands are mapped with hand’s appearance through real-time object detection. The proposed system is verified in a simulation environment through efficiency evaluation with dynamic gestures of the existing vision-based system in addition to usability comparison with traditional joystick controller conducted for applicants with no experience in manipulation. As a result, it proves that it is a safer and more intuitive HUI design with a 0.089 ms processing speed and average lap time that takes about 19 s less than the joystick controller. In other words, it shows that it is viable as an alternative to existing HUI.

## 1. Introduction

### 1.1. Research Backgrounds

In recent years, interest in UAV has increased in civilian applications, such as aerial photography, delivery, transportation, rescue, and surveillance. Naturally, the interaction between humans and UAVs has become frequent, and with the development of artificial intelligence (AI), intelligent HUI approach is actively being developed as an alternative to traditional joystick-based controller [1]. Unlike joystick-based controller, which is limited to highly trained professionals with complex interfaces, the HUI system allows non-skilled users to easily control with the goal of designing a natural and intuitive human-centered interfaces. The field of HUI research is progressing toward designing more innovative and natural interfaces and can be largely classified into four categories: wearable sensors, more user-friendly remote controllers, speech recognition, and hand gesture recognition [2]. The advantages and disadvantages of each HUI are summarized in Table 1. In particular, research on hand-gesture-based recognition (HGR) is being more actively conducted than other research due to the characteristics of hand gestures, which are intuitive and natural means of communication to convey meaningful information between people [1,3,4,5].

### 1.2. Problem Description

According to the related works on HGR, they mainly focus on designing the recognition algorithms with different data sources. Therefore, the HGR can be divided into two categories based on the type of data being processed: the sensor-based gesture recognition (SGR) and the vision-based gesture recognition (VGR) [6]. After that, each approach can be divided in detail according to data collection, pre-processing steps, and training methods, such as machine learning or deep learning [7]. The SGR uses one-dimensional raw data extracted from glove-based wearable sensors composed of multiple sensors, such as IMU, ElectroMyoGram (EMG), and flex sensors, and so on [8,9,10]. The SGR system has low computation, in that it uses relevant data without the process of feature extraction, and it is robust to external environments, such as lighting conditions. However, the majority of studies have been focused on VGR because the VGR system is affordable and easier to collect data than the SGR system. VGR concentrates on analyzing 2D images, 3D images, or video obtained from optical sensors. VGR research can be divided into handcrafted feature-based and deep feature-based approaches.

Compared to handcrafted feature-based approaches, such as Markov models [11,12] and support vector machines (SVMs) [13], which have limitations in gesture complexity and modeling limitations, studies are focused on deep feature-based approaches with relatively low cost by end-to-end training and enhanced feature extraction capabilities. The deep feature-based approaches consist mostly of a process of detecting, recognizing, and interpreting static and dynamic gestures. Here, static gesture means that there is no change in movement for several frames, and dynamic gesture means a combination of poses with various movements across multiple frames [14]. In the case of static gesture recognition, it shows high performance through neural-network-centered approaches, but dynamic gesture recognition still has a challenging problem, in that it is difficult to estimate the pose of a multi-dimensional hand gesture on 2D images. To solve this problem, as in the case of tracking through simple KF of gestures detected through the proposed developed model of Tiny-YOLOv2 [15] and tracking through Deep SORT of skeleton extracted through OpenPose [16], tracking algorithms in addition to deep-learning networks are combined and consequently increase computational complexity. However, complex algorithms are not suitable for the goal of safe and intuitive design of the HUI system, a hard real-time system that values real-time performance.

Therefore, in this paper, we propose a hybrid hand gesture system that combines an IMU-based motion capture system and a vision-based gesture system to increase real-time performance. As an alternative to dynamic gesture recognition in VGR systems that are difficult to estimate pose, gesture recognition can be more easily recognized by utilizing estimated orientation information from IMU-based motion capture system. In the proposed system, the IMU-based control commands are intuitively mapped to be controlled in the same direction as the orientation of the thumb-mounted micro-IMU, and the vision-based control commands are mapped to the hand’s appearance obtained through real-time object detection to perform defined control. The proposed system can be confirmed to be a safe and intuitive interface through usability and efficiency comparisons with traditional joystick controller and existing VGR systems. The main contribution of this paper is as follows:

1. We propose a safer and more intuitive interface by combining IMU-based motion capture system and vision-based system.
2. The proposed system compensates for the disadvantages of sensor-based system: proposal of wearable system.
3. The proposed system compensates for the disadvantages of vision-based system: recognizing complex dynamic gestures using an IMU sensor reduces system complexity and computational amount.

In the subsequent sections, Section 2 presents related work, including two types of hand-gesture-based registration. Section 3 presents the proposed system’s overall architecture. In Section 4, the developed system is presented, followed by static gesture recognition and dynamic gesture recognition. Then, Section 5 discusses the experimental results. Conclusions are drawn in Section 6 and finally, it can be expected to provide a safe and intuitive HUI interface that can be easily operated by non-professionals.

## 2. Previous Research

Various studies have been conducted on the interaction between human gestures and UAVs using different sources of information. The hand gesture recognition methods can be broadly categorized as either SGR or VGR according to the type of data being processed.

### 2.1. Sensor-Based Gesture Recognition Systems

There are many algorithms for SGR. IMU-based SGR can be divided into two parts: data-based and machine-learning (ML)-based. Data-based method utilizes acceleration information extracted from IMU sensors attached to the hand. They provide the valuable information about hand pose, finger joints’ angular pose, and from this we can recognize gestures [17,18]. In addition, in Ref [19], an approach to implement hand motion tracking and recognition with data received from a data glove composed of accelerometers was presented. Most data-based approaches have an advantage in computational complexity to recognize hand gestures. However, there is a disadvantage, namely that it is inconvenient, since the users have to wear sensors. ML-based SGR approach is a method of using machine-learning technology, and there are various classification techniques for gesture recognition algorithms, namely, decision tree (DT) [20], artificial neural networks (ANN) [20,21], K-nearest neighbors (KNN) [22], and SVM [23,24]. In Ref [25], Muezzinoglu, T. compared the results extracted from data gloves for DT, SVM, and KNN classification algorithms. Although ML-based approaches have the advantage of high accuracy, they have the disadvantage of high computation and poor accuracy for data for people who do not undergo training. In addition, research on systems using surface-electromyogram (sEMG) sensors and Flex sensors along with IMU sensors is being actively conducted. In Ref [26], Mardiyanto, R. proposed to control the underwater remote-operated vehicle by mounting the IMU to the elbow, forearm, and wrist, and to control the gripper of the robot by attaching the flex sensor to the finger. In Ref [27], Kim, M.K. presented a real-time motion and force capturing system that combines sEMG with IMU. In the proposed system, the IMU captures arm motion, and the sEMG detects the hand force of human. In the case of such a system, there is a disadvantage, in that mapping is not natural as a time-varying signal that relies on human physical condition.

### 2.2. Vision-Based Gesture Recognition Systems

Hand gesture recognition can be seen as an object detection problem, so VGR algorithms can be divided into handcrafted feature-based and deep feature-based approaches. Handcrafted feature-based algorithm is characterized by training features extracted manually by humans. It has the following pipelines: pre-processing, handcrafted feature extraction, and trainable classifier [28,29,30]. It is less complex than deep learning but has the disadvantage of showing limited performance. Recently, deep feature-based approaches [31,32], such as convolutional neural networks (CNN) and long short-term memory (LSTM) network, have achieved great performance for hand gesture recognition. However, deep feature-based approach for gesture recognition has many aspects to be solved for dynamic hand motion recognition, and various studies are being conducted. In Ref [15], Kassab, M.A. proposed to detect three parts of the interacted person and tracking the detected parts using Kalman filter (KF). In Ref [16], dynamic gesture recognition proceeds with skeleton extraction through OpenPose and human tracking through DeepSORT. In order to improve the efficiency and performance of dynamic gesture recreation, it performs detection and tracking algorithms and consists of high computation and complex systems [2,6].

Therefore, the proposed method to increase the real-time performance, which is important for UAV control, is as follows. The dynamic gestures, which are relatively difficult to recognize in vision-based systems, perform recognition in IMU-based system. Control commands with movement in the same direction as orientation data extracted from IMU mounted on the thumb are intuitively mapped. In addition, static gestures perform mapped control through real-time object detection of vision-based system. Therefore, in this paper, we propose an interface that can be easily controlled by non-professionals by combining the sensor-based system and the vision-based system and recognizing gestures efficiently.

## 3. System Architecture

**Figure 1:**
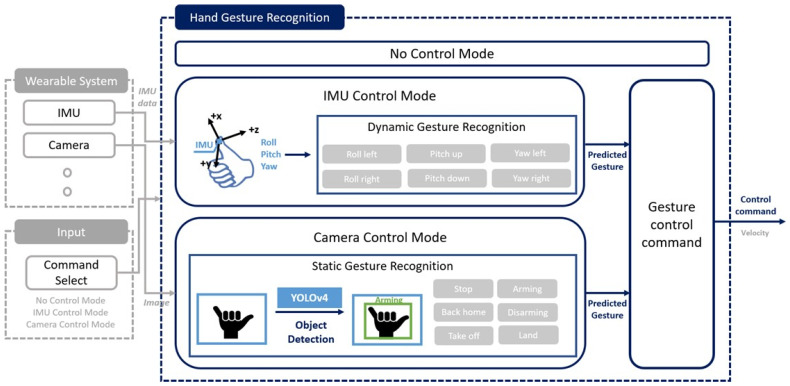
The proposed architecture on hand gesture recognition for controlling UAV.

The objective of the proposed system is to control UAVs with a more natural and intuitive HUI system by efficiently separating gestures in consideration of the characteristics of each sensor. The overall structure of this system consists of two major steps: wearable system and hand gesture recognition system, as shown in Figure 1.

### 3.1. Wearable System

We proposed gesture recognition based on multiple sensors using IMU and camera sensors, and for this purpose, a command system using both hands was implemented. To implement this system, a wearable system and gesture recognition environment were constructed using micro IMU, camera, and thimble for fingers. For dynamic gesture recognition through the IMU, the system with the IMU attached to the operator’s right thumb was designed. For vision-based static gesture recognition, it was configured to wear three-colored thimble to improve real-time object detection performance.

The wearable system is designed based on the command transmission method through both hands. The gesture recognition through IMU can be implemented by attaching an IMU to the operator’s right thumb, and vision-based gesture recognition can be configured to wear three-color thimble to improve the accuracy of real-time object detection. The hand gesture information can be obtained as orientation and image data from the wearable system and the gesture recognition environment.

### 3.2. Hand Gesture Recognition System

The sequential components for hand gesture recognition consist of data collection from wearable system, data processing for gesture recognition of IMU-based motion capture system and VGR system, and calculation of control command values for each recognized gesture. First, the hand gesture information can be obtained as orientation and image data from the wearable system and the gesture recognition environment. Next, each gesture is recognized through real-time object detection or motion estimation algorithm according to each control mode using the gesture information. The control mode is in the form of a state machine, divided into three modes: not control mode, camera control mode, and IMU control mode, which are selected according to the command of the operator. Then, control command according to the recognized gesture is performed to control UAV.

## 4. Motion Capture and Hand Gesture Recognition-Based Real-Time HUI System

### 4.1. Gesture Definition

**Figure 2:**
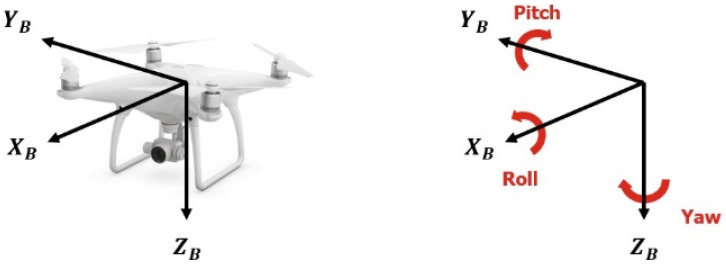
Multi-copter reference coordinate system and coordinate system reference direction.

In order to efficiently classify static and dynamic gestures, the traditional remote controller with a joystick-based multi-copter operation method was considered. Essentially, the movement of the drone consists of translation and rotation based on the three axes, as shown in Figure 2.

**Figure 3:**
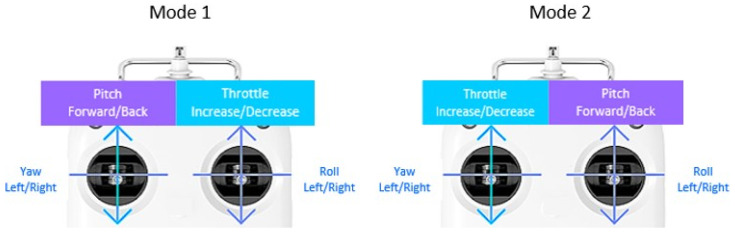
How to control a multi-copter using a twin stick controller.

As can be seen from Figure 3, when rotating in the roll direction through joystick operation, the drone moves left and right, as it tilts left and right. When rotating in the pitch direction, the drone rider moves up and down to move forward or backward. In the case of yaw rotation, the drone is rotated while it is horizontally maintained, and movement against rising and falling is performed by throttle operation. In addition, it can perform various functions, such as arming, disarming, back home, take off, and landing.

For gesture mapping suitable for the proposed gesture recognition-based UAVs control, command lists were selected, as shown in Table 2, in consideration of the joystick-based multi-copter control command. Essentially, it consists of position control in the direction of roll, pitch, and yaw, and altitude control commands of the multi-copter. In addition, *Arming*, *Disarming*, which additionally turns on/off the start of the aircraft motor, and the *Stop* command to stop the movement of the multi-copter and enter the hovering state, were selected. *Take off* and *Land*, which are instructions to take off and land the multi-copter, and *Back home* command to return the multi-copter to its initial takeoff point, were selected.

**Figure 4:**
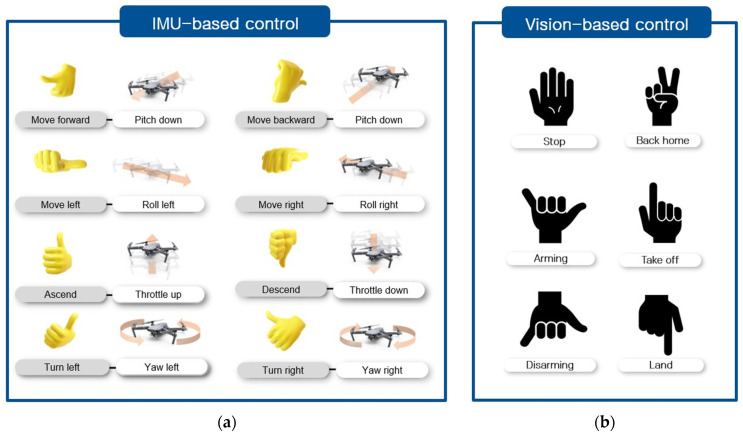
(**a**) Dynamic gestures for IMU-based gesture recognition.; (**b**) Static gestures for vision-based gesture recognition.

The selected commands list can be classified according to the characteristics of the continuous manipulation input. The commands, such as roll, pitch, yaw, and throttle, require the operator to maintain continuous operation input. On the other hand, *Arming*, *Disarming*, *Stop*, *Take off, Land*, and *Back home* commands do not require continuous operation input due to their relatively low frequency of operation input. Therefore, in consideration of the characteristics of continuous manipulation input and frequency of manipulation input, the selected commands can be classified into IMU-based and vision-based gesture recognition commands. In the case of roll, pitch, yaw, and throttle commands, the operation was implemented by mapping to IMU, which has the characteristics of high sampling cycles and intuitive orientation estimation. In the case of *Arming*, *Disarming*, *Stop*, *Take off*, *Land*, and *Back home* commands, the operation was mapped with hand gesture recognition through the camera. When configuring commands, hand gestures that are not frequently used on a daily basis were defined, so that unnecessary recognition was not performed. The mapping between the command and the corresponding control for IMU-based and vision-based gesture recognition system is shown in Figure 4.

### 4.2. Gesture Recognition of IMU-Based Motion Capture System

In the proposed system, the pointing gesture method using IMU mounted on the thumb is used. The control method through the direction indicated by the thumb can be operated in a more intuitive and simple way. It is expected to be easily controlled by non-professionals. The system consists of two steps as follows: (1) Alignment and (2) Orientation estimation.

#### 4.2.1. Alignment

Even if each person makes the same gesture, it can appear in various ways depending on their unique habits and characteristics. Therefore, alignment mode was added before classification. The orientation data on the forward command were collected for 5 s while stationary. A process of setting the average value of the collected orientation to the origin of the reference coordinate system was performed. Therefore, IMU-based gesture recognition estimates a pose based on the reference coordinate system.

#### 4.2.2. Orientation Estimation

**Figure 5:**
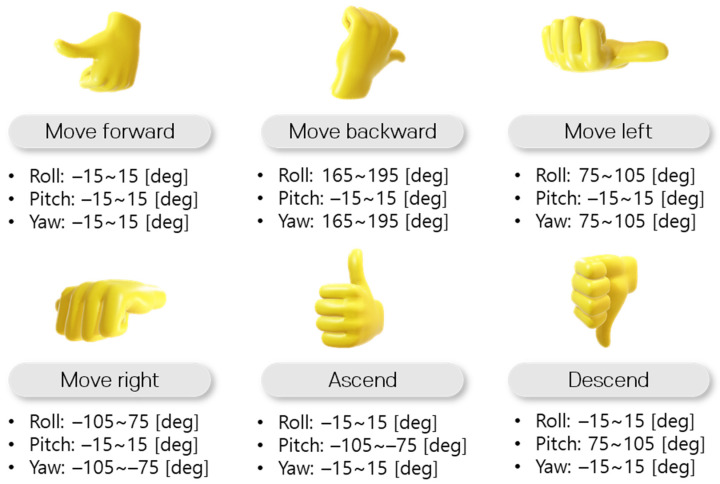
Configuration of the orientation range of the thumb for recognizing each dynamic gesture.

After performing the alignment, the process of recognizing the gesture through the IMU attached to the thumb is performed. The orientation data of the thumb is estimated based on the reference frame, whose *z*-axis points up. Using the orientation extracted from IMU, a corresponding dynamic gesture can be recognized. For example, when the thumb has a pose within 20 degrees left and right on the *x*-axis, 20 degrees up and down on the *y*-axis, and 20 degrees left and right on the *z*-axis, it is recognized as a “*Move forward*” command. When the direction of the thumb is within 20 degrees left and right on the *x*-axis, within 130 to 180 degrees on the *y*-axis, and within 160 to 200 degrees on the *z*-axis, it is recognized as a gesture command of the “*Move backward*” command. In this way, a total of six dynamic gestures according to the orientation of the thumb are defined as shown in Figure 5. Finally, a movement command according to the recognized gesture is performed, and the UAV is controlled.

The proposed mapping method of gestures through IMU-based motion capture system and gestures through VGR system results in an intuitive interaction between the operator and UAVs, which is meaningful, in that the operator does not have to remember most of the designed gesture mapping. For example, when we control UAVs higher than the purpose, we can point our thumb down rather than thinking of complex gesture mapping to make a decision. So, the proposed system can make it easier to control the movement of UAVs through the intuitive HUI when the user observes that the UAVs deviates from the desired path in the precision control area.

### 4.3. Static Gesture Recognition

#### 4.3.1. Dataset Construction

**Figure 6:**
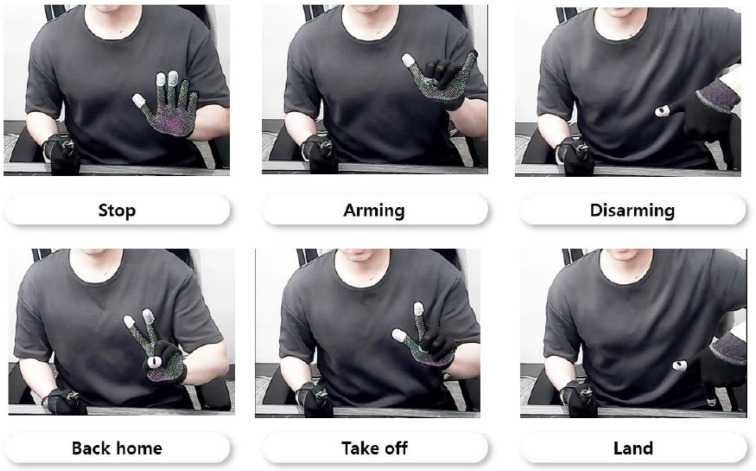
Example of the dataset regarding a gesture definition for each labeled class for vision-based gesture recognition.

In order to recognize gestures through the deep-learning-based model, a class-labeled training dataset for each gesture is essential. Since there are a total of six commands through static gesture recognition that we defined in the previous section, the training dataset we built consists of a total of six classes: *Stop*, *Arming*, *Disarming*, *Back home*, *Take off*, and *Land*, as shown in Figure 6. The gesture for each class was defined as the hand gesture that is not frequently used on a daily basis, so that unnecessary command input is not made when performing operations.

**Figure 7:**
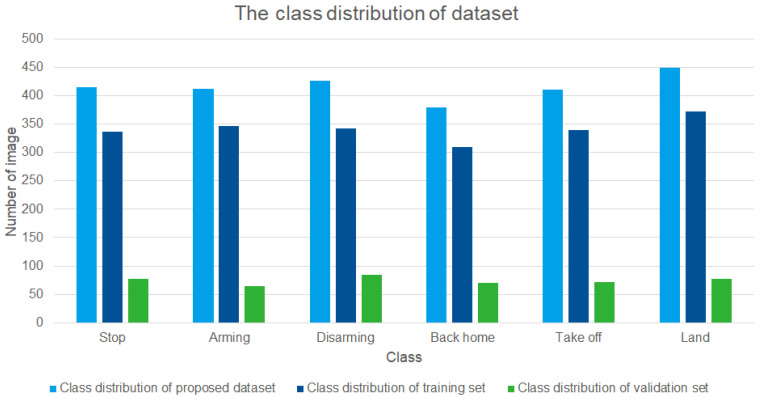
The class distribution of dataset. The *X*-axis represents class, and the *Y*-axis represents the number of images for each class.

The camera sensor to be used in the experiments, oCam-5CRO-U-M, was used to build the dataset. In order to build the dataset, a total of 2494 images of gestures were collected from 9 applicants. For training of the model, the dataset is divided into 2048 images (82.12%) and 446 images (17.88%), respectively, with training and validation set. Figure 7 shows the distribution for each class of the overall data set, training set, and validation set.

#### 4.3.2. Static Gesture Recognition Model

Image-based object detection models can be largely divided into two types: two-stage model and one-stage model. The two-stage model separately conducts bounding boxes prediction and class prediction. On the other hand, a one-stage model predicts bounding boxes and classes at once. In the case of the two-stage model, accuracy is high, but there is a disadvantage in that it takes a long prediction time. In contrast, the one-stage model has the advantage that the prediction time is very fast, although the accuracy is slightly lower than that of the two-stage model. In the case of the gesture recognition for UAV control, real-time performance is the most important factor. Therefore, the proposed system for recognizing static gestures is based on the one-stage model. Among them, YOLOv4 [33] that guarantees high accuracy as well as real-time performance was selected as the static gesture recognition model. As a result of training YOLOv4 using the constructed dataset, YOLOv4 achieved 98.3% mean average precision (mAP) and 91.7% average IoU. Detailed results on the performance of the static gesture recognition model are described in Section 5.2.

**Figure 8:**
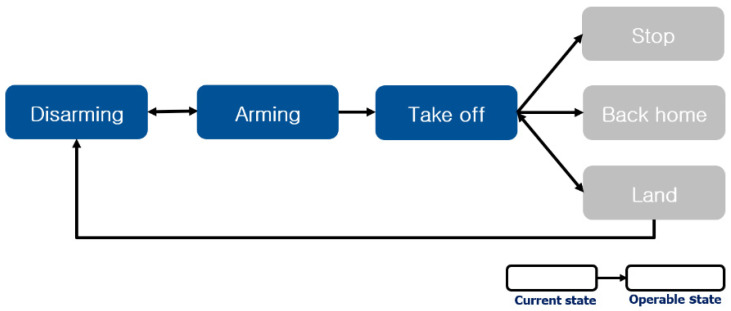
Graph of the relationship between the static gesture commands.

Using the trained YOLOv4 model, a confidence score indicating the probability of prediction of the class can be obtained through static gesture recognition, and a threshold value of 90% can be set to exclude gestures with low confidence score. In addition, it was implemented to prevent unnecessary input of commands by defining the precedence relation between static gesture commands. A state was defined to indicate the states of *Disarming*, *Arming* (or *Land*), and *Take off* in which precedence relationships exist, so that operable commands according to each state were executed. As shown in Figure 8, if the current is *Disarming*, the operable command is *Arming*. When *Arming* (or *Land*) is in the current state, the *Disarming* and *Take off* commands are available. When the current state is *Take off*, it can be seen that the *Stop*, *Back home*, and *Land* commands can be operated.

## 5. Experiments

In order to verify the proposed method, the experiments were conducted focusing on the test of the utility of each function through gesture recognition and the usability in terms of lap time comparison with the traditional joystick-based controller. Considering the safety issue that may occur during UAV control and the experimental scenarios to be described later, the proposed algorithm was evaluated in Gazebo with PX4 [34] software in the loop (SITL). Experiments on the simulation environments were conducted on 10 non-experts who had never controlled the UAVs. Finally, we demonstrated the suitability of the proposed HUI system for non-experts in controlling UAVs.

### 5.1. Experimental Setup

#### 5.1.1. Wearable System Design

**Figure 9:**
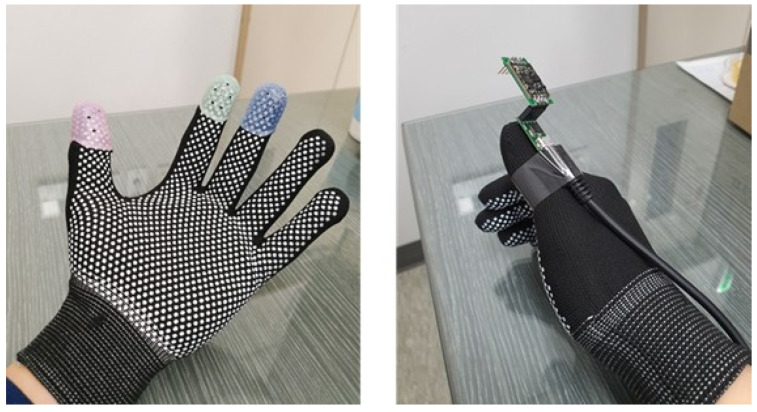
Wearable system for gesture recognition.

Unlike smart gloves with complicated structure, we built a wearable system and gesture recognition environment based on a miniature IMU and camera. This system is based on both hands, and the configuration is as shown in Figure 9. The IMU can be attached to an operator’s right thumb to collect data on the pose and perform gesture recognition. The operator’s left hand performs gesture recognition through real-time object detection of the camera and is configured to wear three colors of thimble to improve detection performance. The model of the IMU sensor used in the system is EBIMU-9DOFV5. It is an ultra-small AHRS module with built-in three-axis accelerometer, three-axis gyroscope, and three-axis magnetometer, and it supports improved sensor precision and improved sensor calibration. In the case of a camera sensor, oCam-5CRO-U m model was used. This has the advantage of being able to link with various software with USB video class (UVC) support and can be used even in low-performance systems, as it is designed to allow video data to be written to memory. Therefore, it was used because it can be used in robot operating system (ROS) environment without installing a separate driver due to UVC compatibility.

#### 5.1.2. Simulation Setup

**Figure 10:**
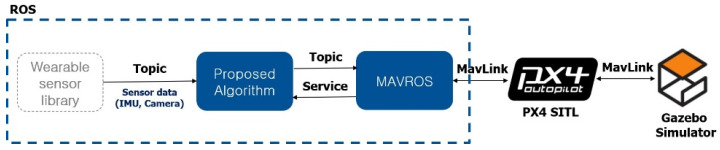
The simulation setup of proposed system.

The simulation environment of the proposed method is based on ROS. Among the various simulators for UAVs control, the experiment was conducted in the Gazebo simulator because it is open source and compatible with ROS. It also sets up SITL, the PX4 firmware used in the simulation, to perform a low level of control over position, attitude, and velocity. This setup can be seen in Figure 10.

The test scenario in the simulation environment was constructed with reference to the practical test for the pilot certification of the ultra-light flying device conducted by Korea Transportation Safety Authority (TS) [35]. Compared to completing 10 h of flight time and conducting practical tests for actual pilot qualification, the operation time of the test using the proposed system is quite insufficient. Accordingly, in the “(1) take off, (2) straight and backward level flight, (3) triangle flight, (4) rhombus flight (rudder turn), (5) crosswind approach, and land” phase of the practical test, the procedures of “(2) straight and backward level flight” and “(4) rhombus flight” were used to construct the segments of the test scenario. In the rhombus flight, the scenario was constructed to manipulate the heading of the UAV toward each destination instead of the rudder turn method. In addition, a segment of “(6) approaching the roof of the building” was constructed, and the entire test scenario can be seen in Table 3.

**Figure 11:**
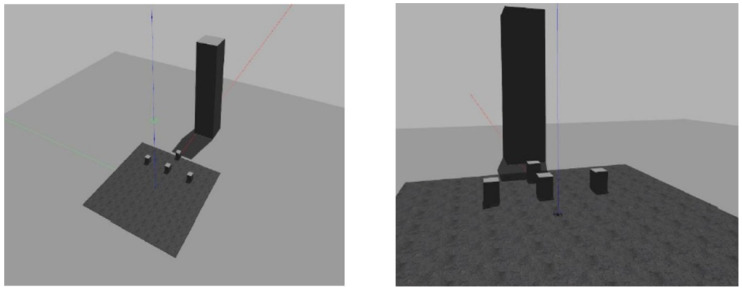
Implementation of simulation environment using the Gazebo.

In order to proceed with the test according to the scenario, the Gazebo environment was established as shown in Figure 11. Four boxes were placed for “(2) straight and backward level flight” and “(4) rhombus flight”, and about 200 m high building was placed for “(6) approaching the roof of the building” segment to establish a test environment that complies with the scenario procedure.

### 5.2. Experimental Results

The experiments on the proposed algorithm consist of three parts: evaluation of VGR system-based gesture classification, evaluation of the efficiency of gesture through IMU-based motion capture system, and evaluation of usability in terms of lap time through comparison of traditional joystick-based controller and proposed interface.

#### 5.2.1. Evaluation of VGR System-Based Gesture Classification

**Figure 12:**
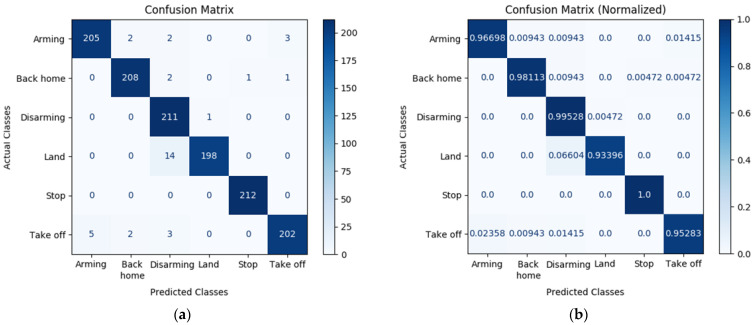
Confusion matrix for trained YOLOv4 model for test set. (**a**) Confusion matrix without normalization.; (**b**) Normalized confusion matrix.

In this section, we conducted a test to confirm the classification performance of static gesture recognition. We used confusion matrix as metric to usefully express the classification performance of the static gestures by referring to the preliminary research on the HGR system [15,16,25,36]. Figure 12 shows the confusion matrix and normalized confusion matrix on the test set, and the diagonal value of the normalized confusion matrix shows high density. This verifies that static gestures are accurately predicted and confirm the functionality of the vision-based system as an interface.

#### 5.2.2. Evaluation of the Efficiency of Gesture through IMU-Based Motion Capture System

In this section, an experiment was conducted on the efficiency of gesture recognition through IMU-based motion capture on the proposed system. Because real-time performance is most important in controlling UAVs, we proposed a method of replacing the dynamic gesture part of the complex vision-based system with IMU-based system. Therefore, we compared the computation time required to operate the commands of the proposed IMU-based system and vision-based system in addition to verifying the utility of each gesture through IMU-based motion capture system.

First, to confirm the functionality of the IMU-based motion capture system as an interface, an experiment was conducted on 10 applicants who had no experience in operating drones. Referring to the evaluation method of related HUI studies [28,37], each applicant conducted an experiment 30 times for each command, and as shown in Table 4, the accuracy was analyzed by counting the number of successes and failures of the commands. Since the command for each gesture was performed without being significantly affected by the applicant, IMU-based motion capture system proved to be suitable as an interface.

Next, the speed of the proposed system and vision-based system for dynamic gesture recognition were compared, which can be seen in Table 5. Chen, B. [2] proposed a six-action gesture system through graph convolutional network (GCN), and it takes 45 ms to recognize the dynamic gesture. In the case of Kasab, Mohamed A. [15], a system for recognizing dynamic gesture through directly developed simplified Tiny-YOLOv2 was proposed. It is performed with KF algorithm and takes 42.7786 ms. Liu, C. [6] proposed two dynamic gesture recognition systems using CNN-based detection, which takes 20 ms.

Finally, unlike the previous vision-based system, we proposed IMU-based dynamic gesture recognition, which takes 0.089 ms to recognize them. Processing time may vary depending on the experimental environment, but considering the data and algorithms that are processed, there will be no significant change in the processing speed. Through this, we can prove that gesture recognition through our proposed IMU-based system is suitable for controlling UAVs.

#### 5.2.3. Evaluation of Usability in Terms of Lap Time

The goal of the proposed system is to design a safe and intuitive interface for easy control by non-professionals, so the test was conducted using lap time, one of the flight performance metrics significantly affected by the type of interface [38,39,40]. It is a test that measures the lap time of a traditional joystick-based controller and a proposed system-based controller, and the experiment was conducted on 10 applicants who had no experience in drone control. At this time, after giving the same practice time, the time taken to start from the starting line to the stop line was measured according to the previously defined scenario. The test result is as shown in Table 6. Even though applicants were given the same practice time, average lap time of the proposed method took about 19 s less than joystick-based controller. The difference in average lap time proves that the proposed system is more intuitive than a joystick-based controller. In other words, an intuitive and natural HUI system was established, and even non-experts can expect to be able to easily control the UAV.

## 6. Conclusions

We propose a real-time human–UAV interaction system that combines an IMU and a camera. Instead of traditional joystick-based control, which requires a lot of training time, it aims to build an intuitive and natural interface for easy control by non-professionals. According to preliminary research, studies are being actively conducted with sensor-based systems and vision-based systems.

However, vision-based systems require a large amount of computation according to complex algorithms in recognizing dynamic gesture. Real-time performance is an important factor in controlling UAVs, and in consideration of this, a hybrid system that combines IMU-based system and vision-based system was proposed. The commands used in the joystick-based controller were defined, and mapping was performed in accordance with the characteristics of each sensor and command. Therefore, by efficiently classifying the gestures of the IMU-based motion capture system and the gestures of the VGR system, it was possible to build an intuitive and natural interface.

The experiment was conducted on 10 non-experts who had never controlled the UAVs to evaluate the classification performance of vision-based gesture recognition, the efficiency of gestures through IMU-based motion capture system, and usability evaluation in terms of lap time by comparing the existing joystick-based control system and the proposed system. First, the classification performance of static gesture recognition was 98.3% mAP and 91.7% average IoU, and the suitability as a static gesture detector was confirmed through the classification performance on the test set. Second, in the evaluation of the efficiency of IMU-based gesture, it showed high performance without performance change, according to the applicants. In addition, the suitability of proposed gesture recognition system was confirmed through computation comparison with the existing vision-based gesture recognition system. Finally, the lap time measurement of the joystick-based system and the proposed system according to a given scenario was also compared. Although the same practice time was given for each interface, it was confirmed that the UAV control through the proposed system completed more quickly, on average by about 19 s. Consequently, it was verified that the proposed system is a safer and more intuitive human-centered design than the VGR system, which has computation complexity. In other words, it shows that it is viable as an alternative to existing HUI, and we expect that it can be easily operated by non-experts.

In future works, we will apply the proposed system in the real world, overcome the sensitivity of operation and environmental conditions, and propose a more robust system by using additional sensors, such as EMG, expanding the scope of gesture commands using cameras, or securing precision in IMU operation. In addition, in terms of interface design, we will design a more intuitive interface using a variety of evaluation metrics, such as safe feeling, satisfaction parameters, and task accumulation. If these points are supplemented, it will be possible to build a UAV command system through more efficient and user-friendly gesture recognition.

## Figures and Tables

**Table 1:** Advantages and disadvantages of four types of categories in the HUI.

HUI	Advantages	Disadvantages
Wearable Sensors	- Intuitive, Natural - Low computation compared to other interfaces - Suitable performance for human motion capture	- Expensive equipment required - Lack of ability to distinguish between unconscious and predefined similar behaviors
More User-Friendly Remote Controller	- Less training time compared to traditional remote controller - Additional features, such as path planning using touch-screen devices	- Less intuitive than other interfaces - Distance limitation by WiFi signal transmission characteristics
Speech	- Intuitive, Natural - No additional devices required	- Decreased performance due to influences from surrounding environment, such as noise - Effects of language and intonation differences
Gesture	- Intuitive, Natural - No additional devices required - No need for many commands	- Lower control performance than other interfaces due to limited discriminant ability - High computation compared to other interfaces

**Table 2:** Command list considering joystick-based multi-copter direct operation instructions and proposed system.

No.	Command	No.	Command
1	Move forward (Pitch down)	8	Descend (Throttle down)
2	Move backward (Pitch up)	9	Arming
3	Move left (Roll left)	10	Disarming
4	Move right (Roll right)	11	Take off
5	Turn left (Yaw left)	12	Land
6	Turn right (Yaw right)	13	Back home
7	Ascend (Throttle up)	14	Stop

**Table 3:** Test scenario procedure.

No.	Segment	Operation
1	IMU alignment	Proceed with IMU alignment in neural mode [N]
2	Arming	Switch to camera control mode [C]
		Perform *Arming* command with vision-based gesture recognition
3	Take off	Perform *Take off* command with vision-based gesture recognition
4	Straight and level flight	Switch to IMU control mode [I]
		Mode home position—C point (5 s waiting) with IMU-based gesture recognition
5	Backward and level flight	Move C point—home position with IMU-based gesture recognition
6	Rhombus flight	Move home position—B point—C point—D point—home sequentially through IMU-based gesture recognition
7	Target approach	Move home position—building structure through IMU-based gesture recognition
8	Back home	Switch to camera control mode [C]
		Perform *Back home* command with vision-based gesture recognition
9	Stop	Perform *Stop* command with vision-based gesture recognition
10	Land	Perform *Land* command with vision-based gesture recognition
11	Disarming	Perform *Disarming* command with vision-based gesture recognition

**Table 4:** Result of evaluating the utility of each function for each dynamic gesture.

	Function	Accuracy
IMU-based gesture command	Move forward	97.78%
	Move backward	97.78%
	Move left	98.89%
	Move right	100%
	Turn right	91.11%
	Turn left	92.22%
	Ascend	96.67%
	Descend	100%

**Table 5:** Result of comparison between the proposed system and the vision-based system for dynamic gesture recognition.

Authors	Interacted System	Deep-Learning Algorithm	Number of Dynamic Gestures	Processing Speed of Dynamic Gesture Recognition (ms)
Chen, B. [2]	UAV	Yes (GNN)	6	45
Kasab, Mohamed A. [15]	UAV	Yes (Developed Tiny-YOLOv2)	10	42.7786
Liu, C. [6]	UAV	Yes (CNN)	2	20
**Ours**	**UAV**	**No** (**IMU-based system**)	**8**	**0.089**

**Table 6:** Lap time comparison of joystick-based systems and proposed systems of 10 applicants according to a given scenario.

	Joystick-Based Control (mm:ss)	Proposed Method (mm:ss)
Participant 1	02:34	02:29
Participant 2	03:01	02:34
Participant 3	02:11	01:59
Participant 4	03:04	02:13
Participant 5	02:14	01:31
Participant 6	02:07	02:19
Participant 7	02:46	02:38
Participant 8	02:54	02:38
Participant 9	02:25	02:02
Participant 10	02:34	02:16
Average	02:35	02:16

## Data Availability

Not applicable.
